# Optimization of Multiple W_1_/O/W_2_ Emulsions Processing for Suitable Stability and Encapsulation Efficiency

**DOI:** 10.3390/foods11091367

**Published:** 2022-05-09

**Authors:** Manuel Felix, Antonio Guerrero, Cecilio Carrera-Sánchez

**Affiliations:** Departamento de Ingeniería Química, Escuela Politécnica Superior, Universidad de Sevilla, 41011 Sevilla, Spain; aguerrero@us.es (A.G.); cecilio@us.es (C.C.-S.)

**Keywords:** droplet size distribution, emulsification, multiple emulsions, polyglycerol polyricinoleate (PGPR)

## Abstract

Double emulsions are a type of multiple emulsions, which can be defined as a multicompartmentalized system where the droplets are dispersed into the continuous phase containing other emulsions. Although double food-grade emulsions have been manufactured, there is a lack of scientific background related to the influence of different processing conditions. This work analyses the influence of processing variables in (W_1_/O/W_2_) double emulsions: passes through the valve homogenizer, pressure applied, lipophilic emulsifier concentration, the ratio between the continuous phase (W_2_) and the primary emulsion (W_1_/O), and the incorporation of xanthan gum (XG) as a stabilizer. The results obtained show that these emulsions can be obtained after selecting suitable processing conditions, making them easily scalable in industrial processes. In terms of droplet size distribution, the input of higher energy to the system (20 MPa) during emulsification processing led to emulsions with smaller droplet sizes (D_3,2_). However, more monodispersed emulsions were achieved when the lowest pressure (5 MPa) was used. As for the number of passes, the optimal (emulsions more monodispersed and smaller droplet sizes) was found around 2–3 passes, regardless of the valve homogenizer pressure. However, emulsions processed at 20 MPa involved lower encapsulation efficiency (E_E_) than emulsions processed at 5 MPa (87.3 ± 2.3 vs. 96.1 ± 1.8, respectively). The addition of XG led to more structured emulsions, and consequently, their kinetic stability increased. The results obtained indicated that a correct formulation of these W_1_/O/W_2_ double emulsions allowed the optimal encapsulation of both hydrophilic and lipophilic bioactive compounds. Thus, the development of food matrices, in the form of multiple emulsions, would allow the encapsulation of bioactive compounds, which would result in the development of novelty food products.

## 1. Introduction

Functional food products are based on the presence of functional ingredients (bioactive compounds) in the food matrix; the possibility of developing this new generation of food products requires the employment of strategies able to preserve the desired components during their storage and after their intake thorough the intestinal tract [1]. One approach for the development of these functional foods includes the use of current technologies applied to emulsion-based products, making them a reliable alternative to the current food market [2]. In this way, the most versatile path to modify the composition of food arises from the possibility of introducing bioactive ingredients during its preparation [3]. Based on the solubility of these ingredients, they could be classified into two big families: lipophilic and hydrophilic compounds [4]. The former ingredients (e.g., carotenoids, E-vitamin, etc.) are soluble in the oil phase of conventional emulsions, although their encapsulation in nanoemulsion-based systems has been demonstrated to improve not only the physical stability but also their absorption after intake [3]. However, hydrophilic components (e.g., polyphenols, peptides, etc.) cannot be included in the continuous phase of conventional emulsions since their biodisponibility is reduced after digestion [5]. An alternative for introducing these bioactive components in food matrices is to include them in the primary emulsion of double emulsions, protecting them from chemical changes during digestion [6,7].

In this context, the development of double emulsions as food matrices opens up new possibilities in the design and development of functional foods [8]. These food matrices could be used as carriers of water-soluble bioactive components. However, technological strategies must be applied to adapt current food processing methods in the development of this type of emulsion, optimizing the presence of bioactive compounds [8].

Double emulsions are a type of multiple emulsion, which can be defined as a multicompartmentalized system where the droplets disperse into the continuous phase containing other emulsions [9]. These systems are characterized by the coexistence of oil-in-water (O/W) and water-in-oil (W/O) emulsions, in which the dispersed phase (in the form of droplets) is the continuous phase of the primary emulsion (which means that it has smaller droplets of the other phase disperse into it) [10]. Thus, the most common case is water-in-oil-in-water emulsions (W_1_/O/W_2_), although those of oil-in-water-in-oil (O_1_/W/O_2_) can also be used in specific applications (i.e., they exhibit a fine texture, showing a smooth touch upon application) [6,11]. Once produced, double emulsions are subjected to several coalescence and diffusion phenomena that consequently impact product properties, such as texture or encapsulation performance [12].

The use of multiple (double) emulsions in food matrices brings unexplored opportunities to introduce bioactive compounds in food products and, consequently, in human consumption habits. Although double food-grade emulsions have been already manufactured, there is a lack of scientific background related to the influence of different processing conditions.

The general objective of this work is to analyze the influence of processing variables that affect the development of double emulsion (W_1_/O/W_2_). The encapsulation efficiency (E_E_, parameter to be optimized) was assessed by tartrazine, whose use as a marker was initially proven. The different variables studied were: passes through the valve homogenizer, pressure applied, lipophilic emulsifier (polyglycerol polyricinoleate, PGPR), the ratio between the continuous phase (W_2_) and the primary emulsion (W_1_/O), and the incorporation of a hydrocolloid (xanthan gum) as a stabilizer of the double emulsions generated. 

## 2. Materials and Methods

### 2.1. Materials

The lipophilic surfactant polyglycerol polyricinoleate (PGPR) was supplied by Campobetica (Malaga, Spain). This emulsifier was used for the stabilization of primary emulsions (W_1_/O). BiPro^®^ whey protein isolate (WPI), supplied by AGROPUR (Longueuil, QC, Canada), was the emulsifier used for the stabilization of secondary emulsions (W_1_/O/W_2_). Xanthan from Xanthomonas campestris was provided by Merck (Branchburg, NJ, USA). Chemical reagents (i.e., HCl, NaOH, NaCl, Tartrazine and Trizma) were purchased from Sigma–Aldrich company (St. Louis, MO, USA). The oil used (sunflower oil) was purchased from a local market. Emulsions were prepared using deionized water.

### 2.2. Methods

#### 2.2.1. Preparation of W_1_/O/W_2_ Double Emulsions

Firstly, the primary W_1_/O emulsion was prepared. For these emulsions, the aqueous phase was stabilized at pH 7.0 using Trizma (50 mM). NaCl was added for ionic-strength control (50 mM), and tartrazine was used as a marker (0.06 wt.%). This aqueous phase (W_1_) was dispersed into the continuous oily phase, which contained PGPR (1, 3 or 5 wt.%). The W_1_/O ratio of these primary emulsions was, in all cases, 20/80. This primary emulsion was prepared by applying high energy to obtain small-sized droplets. Thus, the oil and the W_1_ phase were blended and mixed using a rotor-stator homogenizer (Utraturrax^®^ T-25, IKA, Staufen, Germany) at 8000 rpm. Subsequently, the emulsion obtained was passed through a high-pressure homogenizer at 50 MPa (Emulsiflex 2000-CS, Avestin, Ottawa, ON, Canada). Once primary emulsions were prepared, secondary emulsions were generated by mixing the primary emulsion with an aqueous WPI solution (0.5 wt.%) at pH 7, W_2_. In order to adjust the osmotic pressure balance between the aqueous phases W_1_ and W_2_, NaCl was also added to this outer aqueous phase. Soft processing conditions (i.e., low energy input) were used in this case to avoid the breakdown of primary emulsions. The W_1_/O primary emulsion was mixed with the outer phase (W_2_) using the same rotor-stator mixer, but at 1000 rpm. These pre-emulsions were also passed through the Emulsiflex valve homogenizer, selecting, in this case, 5 and 20 MPa. The (W_1_/O)/W_2_ ratios of multiple emulsions were 10/90, 25/75 and 40/60. These double emulsions were prepared according to the method proposed by Lynch [13]. Moreover, the influence of the addition of xanthan gum (XG) as a stabilizer was also tested. These emulsions were prepared following the same procedure described above with a (W_1_/O)/W_2_ ratio of 40/60 (processed in absence of XG). Once emulsions were generated (using 5 wt.% PGPR according to the highest EE value), XG dissolved into W_2_ was added up to reach the (W_1_/O)/W_2_ ratio of 25/75. XG solution was dispersed into (W_1_/O)/W_2_ emulsions by soft magnetic stirring. The final XG concentration studied was 0.125 and 0.25 wt.%. Supplementary Table S1 summarizes the systems performed in this research.

### 2.3. Characterisation of Emulsions

#### 2.3.1. Droplet Size Distribution

Droplet size distribution (DSD) of the double emulsions (W_1_/O/W_2_) was determined with the particle size analyzer based on laser diffraction Mastersizer 2000 (Malvern, UK). To facilitate the rupture of possible flocs, 0.5 mL of emulsion samples were diluted in a 1% SDS (sodium dodecyl sulfate) solution. Immediately after this, the droplet size distribution of the deflocculated systems was determined [14]. The mean Sauter diameter (D_3,2_) and volumetric diameter (D_4,3_) were calculated according to Equations (1) and (2):

(1) $$D_{3,2}=\frac{\sum^{\;}n_{i}d_{i}^{3}}{\sum^{\;}n_{i}d_{i}^{2}}$$

(2) $$D_{4,3}=\frac{\sum^{\;}n_{i}d_{i}^{4}}{\sum^{\;}n_{i}d_{i}^{3}}$$

where $n_{i}$ is the number of droplets that have $d_{i}$ diameter.

The span parameter (Equation (3)) was calculated to analyze the DSD profiles:

(3) $$\mathrm{Span}=\frac{d\left(90\right)-d\left(10\right)}{d\left(50\right)}$$

where d(90), d(10) and d(50) refer to the 10, 50 and 90 volume percentile of droplets with diameters smaller or equal to these values.

The Flocculation Index (FI, Equation (4)) was calculated to study the flocculation phenomena occurring during emulsion storage:

(4) $$\mathrm{FI}\;\left(\%\right)=\frac{\left|D_{4,3}-D_{4,3\;\left(\mathrm{SDS}\right)}\right|}{D_{4,3}}\times 100$$

where $D_{4,3}$ and $D_{4,3\;\left(\mathrm{SDS}\right)}$ are the volumetric diameter without and with SDS solution. 

#### 2.3.2. Rheological Properties

Linear viscoelastic properties were analyzed by means of small amplitude oscillatory shear (SAOS) tests using the AR-2000 rheometer (TA Instruments, New Castle, DE, USA). All measurements were carried out within the linear viscoelastic range (LVR), which was determined by means of strain sweep tests. These tests were performed at 1 Hz and from 0.01 to 10% strain. Mechanical spectra were obtained from frequency sweep tests (from 0.1 to 30 Hz) at a constant strain (1%). These tests were carried out using a low inertia aluminum smooth plate (60 mm) at a gap of 1 mm. The temperature was fixed at 25 °C by a peltier system 

#### 2.3.3. Emulsion Stability

The overall stability of emulsions was assessed using a vertical scan analyzer Turbiscan MA 2000 (Formulaction, Toulouse, France) through backscattering measurements of a pulsed light source (λ = 850 nm) as a function of the height of the cylindrical glass tube containing the sample [15]. Emulsions were stored at 5 °C and measured at room temperature. Then, the profiles of backscattering of light from emulsions (ΔBS/100%) versus the height were plotted as a function of storage time.

### 2.4. Encapsulation Capacity

#### 2.4.1. Measurement of Marker Concentration in the Outer Aqueous Phase (W_2_)

One of the most important steps in the determination of the marker concentration is to ensure that the concentration determined for the marked is not affected by the presence of the protein used, the processing conditions or the storage time. In this section, experiments were carried out to ensure the correct measurement of the marker in the final double emulsions [16].

The first determination consisted of checking the correct determination of the marker (tartrazine) in the presence of a WPI solution. The marker was dissolved in Trizma buffer (50 mM, pH 7) at the same concentration as it was added to the inner aqueous phase (W_1_) in the double emulsion (0.06 wt.%). Subsequently, this solution was added to the outer aqueous phase W_2_ at the same ratio as in the final double emulsions. The W_2_ phase contained 0.5 wt.% WPI protein since this is the protein concentration used in the W_2_ of the final double emulsions. W_1_ + W_2_ were mixed at room temperature for 30 min using a magnetic stirrer. The concentration of the marker in the overall solution was calculated by measuring the absorbance at 435 nm of the corresponding solution using a spectrophotometer (Thermo Scientific, Waltham, MA, USA), using the WPI in Trizma buffer as the blank reference. Tartrazine solution was also added to a Trizma buffer solution (without WPI) and treated, but in this case, using the buffer solution as the blank reference. The value of absorbance obtained was interpolated in a calibration curve obtained after dissolving different known concentrations of the marker in Trizma buffer at pH 7 (W_1_).

Measurements were carried out on the same day of W_1_ + W_2_ mixing and after 7 storage days in an oven at 45 °C. The marker measurable concentration ($MC_{m}\left(\%\right)$) in the WPI solution was calculated according to the Equation (5):

(5) $$MC_{m}\left(\%\right)=\frac{C_{m_{WPI}}}{C_{m_{b}}}\times 100$$

where $C_{m_{WPI}}$ is the concentration of the marker measured in the W_1_ + W_2_ (with WPI) mixtures and $C_{m_{b}}$ is the concentration of the marker according to the dilutions carried out (without WPI).

#### 2.4.2. Measurement of the Recovery Yield (Ry) of Marker

The recovery yield is defined as the concentration of marker used, which is present in the aqueous phase recovered after separating the cream phase and an aqueous phase by centrifugation. The percentage of recovery yield is relative to the concentration of marker in the outer aqueous phase after emulsion preparation [16].

For these measurements, a conventional oil-in-water emulsion (O/W_2_) was prepared using the same oil phase (O) and the same outer aqueous phase (W_2_) as in the W_1_/O/W_2_ double emulsion (ratio 10/90). The oil phase (O) was gradually added to the aqueous phase (W_2_) while they were mixing using an Ultraturrax^®^ homogenizer (IKA, Staufen, Germany) at 9500 rpm and 1 min. Subsequently, the pre-emulsion was passed through a high-pressure homogenizer (Emulsiflex 2000-CS, Avestin, Ottawa, ON, Canada). These O/W_2_ conventional emulsions were diluted in the W_1_ phase (reaching the ratio eventually used in W_1_/O/W_2_ double emulsions). The W_1_ contained either (i) the marker at 0.06 wt.% for the determination of marker recovery or (ii) without the marker for the sample blank.

According to Dickinson et al. [17], this procedure ensured that the marker was completely present in the outer aqueous phase. Consequently, this emulsion can be used as the standard where 100% of the marker was present in the outer aqueous phase (W_2_). The determination of aqueous phase color requires a transparent medium. Thus, an aliquot of the emulsions was diluted with the W_1_ phase (1:1), the mixture was homogenized by a soft stirring and, subsequently, it was separated by centrifugation (15,000× *g* at 4 °C for 30 min) in a cream phase (which contained the oil droplets) and an aqueous phase. The aqueous phase was filtered (0.2 μm syringe microfilter), and the concentration of the marker presented in the resulting filtered aqueous phase was calculated by the measurement of the absorbance at 435 nm in a spectrophotometer (Thermo Scientific) using the subnatant obtained from O/W_2_ emulsion with added Trizma buffer without the added marker as a reference blank.

The value of the absorbance obtained was compared with the concentration of the marker in the outer aqueous phase (which depends on the ratio (W_1_/O)/W_2_). The recovery yield can be calculated according to Equation (6) [16]:

(6) $$\mathrm{Ry}\;\left(\%\right)=\frac{C_{m_{OAP\left(1,7\right)}}}{C_{m_{b}}}\times 100$$

where $C_{m_{OAP\left(1,7\right)}}$ was the concentration of the marker used present in the outer aqueous phase (recovered after centrifugation) after 1 and 7 days of emulsion aging. $C_{m_{b}}$ was the concentration of the marker used in the outer aqueous phase. This latter concentration was determined by an equivalent dilution of the aqueous marker solution with Trizma buffer as occurred in the emulsion.

The concentration of the marker present in the outer aqueous phase was calculated on the same day of emulsion preparation and 7 days later to determine the influence of aging on the Ry parameter.

#### 2.4.3. Encapsulation Efficiency (E_E_) Measurement

The E_E_ of the double W_1_/O/W_2_ emulsion was calculated by the determination of the concentration of the marker in the outer aqueous phase (W_2_) just after the preparation of the emulsions.

The E_E_ of double W_1_/O/W_2_ emulsions was prepared according to the experimental procedure indicated in Section 2.2.1. Once double emulsions were prepared, they were subjected to the phase separation explained in Section 2.4.2. Double emulsions were prepared with the marker (tartrazine) diluted in the W_1_ and in the absence of the marker to obtain the blank for the measurement of the absorbance of the aqueous phase. The concentration of the marker in the outer aqueous phase (W_2_) was measured by the same method as described above for measuring the recovery yield but replacing the O/W_2_ emulsion with the W_1_/O/W_2_ emulsion. The E_E_ (%) was calculated with respect to the concentration of the marker present in the aqueous phase obtained after emulsion separation (W_1_ + W_2_) according to Equation (7):

(7) $$E_{E}\left(\%\right)=100-\frac{C_{m_{OAP}}}{C_{m_{b}}}\times 100$$

where $C_{m_{OAP}}$ is the marker concentration measured in the aqueous phase (which has been recovered from the double emulsions), $C_{m_{b}}$ is the marker concentration initially added to the inner aqueous phase.

The E_E_ was calculated on days 0 and 15 to determine the influence of storage time on this parameter.

### 2.5. Statistical Analysis

Emulsions were prepared in duplicate. Three replicates were carried out for each measurement. Significant differences were determined by performing ANOVA tests using STATGRAPHICS Centurion XVIII software version number (Statgraphics Tecnologies Inc., The Plains, VA, USA).

## 3. Results and Discussion

### 3.1. Evaluation of Tartrazine as a Marker for Double Emulsions Stabilized by WPI

The effectiveness of tartrazine as a marker for encapsulating hydrophilic compounds in W_1_/O/W_2_ double emulsions must be addressed before any experiment. To this end, it is essential to identify a marker added to the internal aqueous phase (W_1_), whose concentration in the outer aqueous phase (W_2_) can be determined after double emulsion preparation. Moreover, changes in the concentration of the marker should not be elucidated in the outer aqueous phase during double emulsion storage [16].

Table 1 shows the marker measurable concentration (${\mathrm{MC}}_{m}$) and recovery yield (Ry) for the marker used in this work (tartrazine) in the aqueous phase recovered when 100% of the marker is in the outer aqueous phase (W_2_). Table 1 indicates that the marker used can be accurately measured in the external aqueous phase (W_2_) of the double W_1_/O/W_2_ emulsion by measuring the absorbance of the outer aqueous phase following the procedure indicated in Section 2.4 since the marker concentration measurable (MC_m_) reached 100 ± 0.5%. Moreover, there are no changes in the concentration of tartrazine after 7 days of external phase (W_2_) storage containing tartrazine at 45 °C (MC_m_ = 98.8 ± 0.2%). 

As for the recovery yield (Ry), this table also reveals that the marker selected (tartrazine) can be used for the evaluation of this parameter since the 95.5 ± 1.5% tatrazine remains in the inner aqueous phase (W_1_) the same day emulsion preparation, and 98.9 ± 0.2% after 7 days, where no significant differences were found (*p* < 0.05). These results also show that the tartrazine marker does not preferentially migrate with the cream phase formed after emulsion centrifugation. The high level of marker present in the recovered aqueous phase after centrifugation suggests that tartrazine is equally distributed throughout the aqueous phase of the entire emulsion.

These results indicate that tartrazine does not associate with WPI and that this marker can be used for the evaluation of emulsion efficiency (E_E_) for the double W_1_/O/W_2_ emulsion generated hereinafter. These parameters were more suitable than the values found when methylene blue and B_12_ vitamin were used as markers in double emulsions stabilized by sodium caseinate [16].

### 3.2. Processing Conditions, Optimization Passes and Valve Homogenizer Pressure

#### Droplet Size Distribution Measurements

The ratio 25/75 was used for double emulsions ((W_1_/O)/W_2_) during the optimization of the number of passes through the high-pressure homogenizer (from 1 to 5). The homogenization pressures evaluated were 5 and 20 MPa. The PGPR concentration used for these emulsions was 5 wt.%.

**Figure 1:**
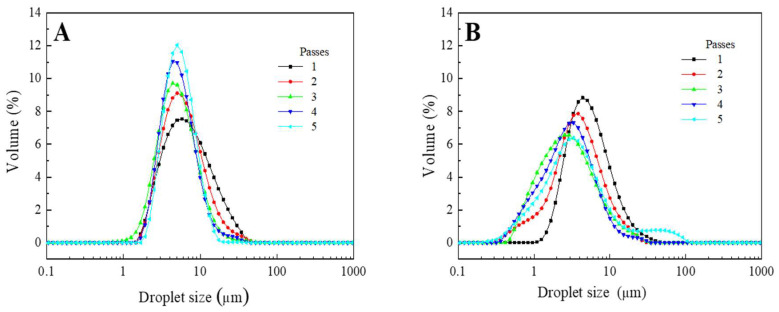
Droplet size of W_1_/O/W_2_ multiple emulsions obtained after processing the secondary emulsion at 5 MPa (**A**) and 20 MPa (**B**), as a function emulsion passes through the high-pressure homogenizer.

Figure 1 shows the droplet size distribution (DSD) of the W_1_/O/W_2_ emulsions obtained after processing the secondary emulsion at 5 MPa (A) and 20 MPa (B) as a function of emulsion passes through the valve homogenizer. All measurements plotted were carried out in the presence of 1% SDS (deflocculating agent) to avoid DSD influenced by the bridging flocculation phenomena [14]. When the homogenization pressure was 5 MPa (Figure 1A), unimodal DSD profiles were obtained in all cases; however, the distribution becomes narrower as emulsion passes increased. Thus, the distributions always started around 2 μm; however, the highest droplets moved from c.a. 40 to 20 μm, where the reduction of droplet sizes most important took place after passes 1 and 2. This reduction in droplet size can be expected since there is an increase in the energy input during emulsion processing as the number the passes increases [18].

On the other hand, when the homogenization pressure increased up to 20 MPa (Figure 1B), the unimodal DSD was obtained only for one emulsion passing through the valve homogenizer. A higher number of passes involved the emergence of other tenuous droplet populations at the ends or the beginning of the main peak. Thus, for passes 2 and 3, shorter droplets were obtained due to the above-mentioned increase in energy input. However, this tendency changed for passes 4 and 5, where higher droplet populations were found. This behavior has been related to excessive processing energy, leading to a higher coalescence rate after passing the emulsion through the valve homogenizer [19]. In this sense, a comparison of Figure 1A,B show that an increase in pressure leads to a shift of the droplet size distribution profiles towards smaller sizes, although giving rise to apparently wider distributions. Moreover, an excess of energy (caused by the number of passes) led to an increase in droplet sizes due to coalescence. A similar influence of valve homogenizer pressure was found by Floury et al. [18] for conventional emulsions stabilized by whey protein isolate.

In order to quantify the tendencies observed by droplet size distributions after processing the emulsions at two different homogenizer pressures (5 and 20 MPa) and passes (up to 5). Table 2 summarizes the Sauter mean diameter, the span parameter and the flocculation index (D_3,2_, span and FI, respectively) for all emulsions studied in this section. The values observed in this Table confirm the results previously observed in the drop size distribution curves since an increase in the number of steps led to a significant decrease in Span (higher droplet polydispersity). As for the D_3,2_, the increase in passes involved a decrease in this parameter, especially for the multiple emulsions processed at 5 MPa. However, more than three passes did not involve significant differences (*p* < 0.05).

The FI parameter accounts for the electrostatic interactions of oil droplets. Flocculation can modify the rheological behavior of emulsions, creating a network that prevents phase separation when highly concentrated emulsions are prepared. However, it can also promote droplet coalescence. In general, low flocculated emulsions were obtained. The standard deviation obtained in this parameter suggests that this flocculation was not too strong, and the system was deflocculated/flocculated by the simple mechanical agitation when the measurement was performed [20].

Thus, in terms of droplet size distribution, the input of higher energy to the system (20 MPa) during emulsification processing led to emulsions with smaller droplet sizes (D_3,2_). However, more monodispersed emulsions were achieved when the lowest pressure (5 MPa) was used. As for the number of passes, the optimal (emulsions more monodispersed and smaller droplet sizes) was found around 2–3 passes, regardless of the valve homogenizer pressure.

The processing parameters selected were also based on the E_E_ of the multiple emulsions generated. In fact, the objective of the present work is to maximize this key parameter. Thus, Table 3 shows the E_E_ for emulsions as a function of the number of emulsion passes through the high-pressure homogenizer and the two pressures studied (5 MPa and 20 MPa). This table evidences a clear reduction in the E_E_ when the homogenization pressure increases. In this sense, the encapsulation pressure was higher when emulsions were processed at 5 MPa than when they were processed at 20 MPa (i.e., 96.1 ± 1.8 vs. 87.3 ± 2.3 for three passes).

No significant differences (*p* < 0.05) were found when the emulsions were processed at the lower pressure (5 MPa). On the other hand, when emulsions were processed at the highest pressure (20 MPa), a significant decrease in E_E_ with the number of passes was found. However, this tendency was not clear for the emulsions processed at this pressure. Schuch et al. [21] evaluated the influence of processing conditions on the E_E_ in double emulsions. They found that the E_E_ directly depended on the size of W_1_/O (primary emulsions) since the bigger the primary emulsion droplets, the higher the encapsulation efficiency. However, an excess of energy in secondary emulsions led to a decrease in this parameter.

From these results, and their comparison with the results obtained from droplet size distributions, hereinafter, the emulsion will be processed at 5 MPa and three passes through the high-pressure homogenizer. These conditions generated emulsions with a high encapsulation efficiency, at the same time as the droplet size profiles obtained were unimodal, which has been related to higher emulsion stability than polydispersed droplet size distributions [22].

### 3.3. Processing Conditions Optimization, Influence of PGPR

#### Droplet Size Distribution Measurements

The optimization of PGPR concentration in double emulsions was carried out using emulsions containing 25/75 of the (W_1_/O)/W_2_ ratio. Moreover, according to the previous results obtained, the pressure in the valve homogenizer was 5 MPa, and emulsions were passed through it three times.

**Figure 2:**
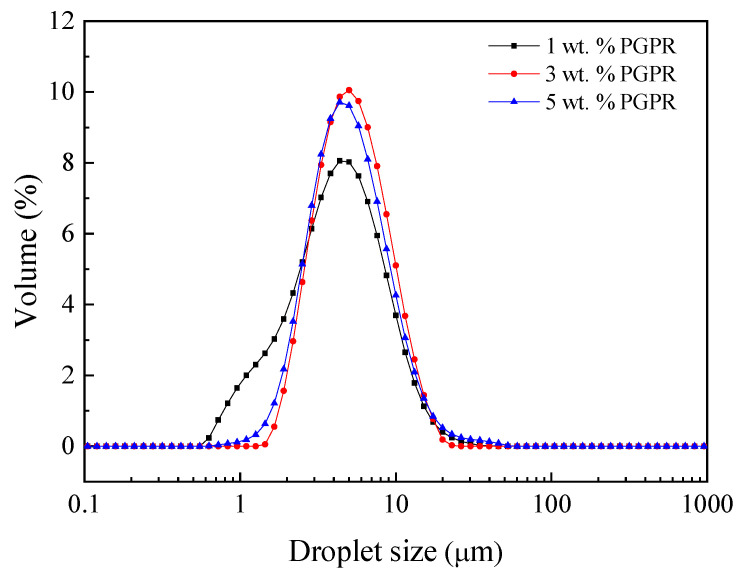
Droplet size distribution of W_1_/O/W_2_ multiple emulsions obtained after using 1, 3 and 5 wt.% PGPR for the primary emulsion.

Figure 2 shows the droplet size distribution of W_1_/O/W_2_ multiple emulsions obtained after using 1, 3 and 5 wt.% PGPR for the primary emulsion. All measurements were carried out in the presence of 1% SDS. The DSD profiles reveal practically single-mode curves, exhibiting a maximum value around 5 µm. In the case of 3% and 5% of PGPR, the curves obtained were practically the same. However, the system containing 1% of PGPR showed a widening in the 1 µm range, resulting in a greater droplet dispersion.

Narrower distributions are desired in emulsions since it determines the potential destabilization processes (such as coalescence and further creaming) [23,24]. This change in DSD profile was also obtained by Altuntas [25] for double emulsions stabilized by PGPR–lecithin mixtures, which evidences the importance of PGPR in double emulsions.

Table 4 summarizes parameters from droplet size distribution (D_3,2_, Span and FI) as well as E_E_ to evaluate the influence of PGPR in these emulsions. This table indicates that the lowest D_3,2_ was obtained for the system containing 1 wt.% PGPR. However, this system also led to the highest Span value (higher polydispersity). Eisinaite et al. [7] found similar results; they observed that the initial droplet size of the double emulsions first increased with the increase in the amount of PGPR and then decreased. They suggest that this behavior could be a result of a combined effect: at higher concentrations of PGPR, the water transfer capacity was higher and swelling was promoted, while the interfacial tension of the droplets was lower, which leads to less swelling.

Moreover, the flocculation index (FI) calculated for these freshly prepared emulsions indicates that it decreased as the PGPR concentration increased. This result is not desired since flocculation phenomena can increase the viscosity of concentrated emulsions; however, in diluted emulsions, it can conduct droplet coalescence [26].

This Table also indicates that a decrease in PGPR led to a significant decrease in E_E_, finding the best results for the emulsion containing 5 wt.% PGPR (96.1 ± 1.8%). These results agree with other studies where double W_1_/O/W_2_ emulsions were prepared with various concentrations of PGPR (0.5–5% wt.%) in the oil phase. Eisinaite et al. [7] found that the emulsions showed good physical stability, with encapsulation efficiency close to 100% only at high concentrations of PGPR (> 2 wt.%). According to these results, the PGPR selected for the further emulsions manufactured was 5.0 wt.%

### 3.4. Processing Conditions Optimization, Influence of (W1/O)/W2 Ratio

Hereinafter, double emulsions were operated under the following operating conditions: low pressure for the valve homogenizer in the final double emulsion (5 MPa), three passes through it and 5 wt.% PGPR in the oil phase for the primary emulsion (W_1_/O). This section analyzes the ratios 10/90, 25/75 and 40/60 between the primary emulsion W_1_/O and the aqueous phase of the secondary emulsion, W_2_, as a function of the aging time.

#### 3.4.1. Droplet Size Distribution and Backscattering Measurements

**Figure 3:**
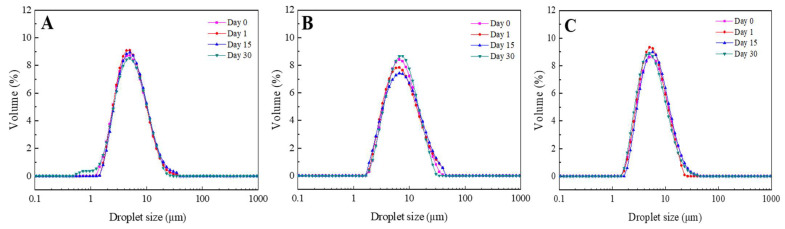
Droplet size distribution of W_1_/O/W_2_ multiple emulsions obtained after three different O/W ratios for the secondary emulsion: 10/90 (**A**), 25/75 (**B**) and 40/60 (**C**).

Figure 3 shows the DSD profiles obtained for the W_1_/O/W_2_ multiple emulsions for three different O/W ratios when the secondary emulsion is prepared: 10/90 (Figure 3A), 25/75 (Figure 3B) and 40/60 (Figure 3C). Moreover, the droplet stability of the emulsions prepared over storage time was also analyzed. All measurements plotted were carried out in the presence of 1% SDS. The DSD profiles obtained for all double emulsions generated were fairly similar, obtaining a unimodal distribution and the droplet sizes were within 2 and 20–30 µm. Moreover, these DSD profiles remained unaltered as a function of storage time, indicating high stability of the three ratios analyzed (10/90, 25/75 and 40/60) over storage time.

In order to evaluate the evolution of the droplet size of the emulsions, Table 5 shows parameters from DSD measurements (D_3,2_, span and FI) of W_1_/O/W_2_ multiple emulsions obtained after evaluating three different O/W ratios for the secondary emulsion (10/90, 25/75 and 40/60) over 30 days storage time. These parameters did not vary significantly over time, where the FI obtained indicates poorly flocculated emulsions. These results suggest that suitable emulsions with the desired stability were obtained regardless of the (W_1_/O)/W_2_ ratio, indicating that the WPI concentration used in the W_2_ phase (0.5 wt.%) was appropriate for the stabilization of the double emulsions [27].

Moreover, comparing the three systems between them, it can be concluded that the emulsion with the lowest proportion of primary emulsion (10/90) has the lowest Sauter mean diameter (D_3,2_). Thus, as the proportion of primary emulsion increased, the droplet sizes increased slightly, finding the highest D_3,2_ values and the wider DSD for the 40/60 system. With regard to these results, it can be found in the literature that an increase in the dispersed phase comes hand-in-hand with increasing the droplet size [28,29,30], as long as the emulsifier concentration and emulsification energy remain constant.

**Figure 4:**
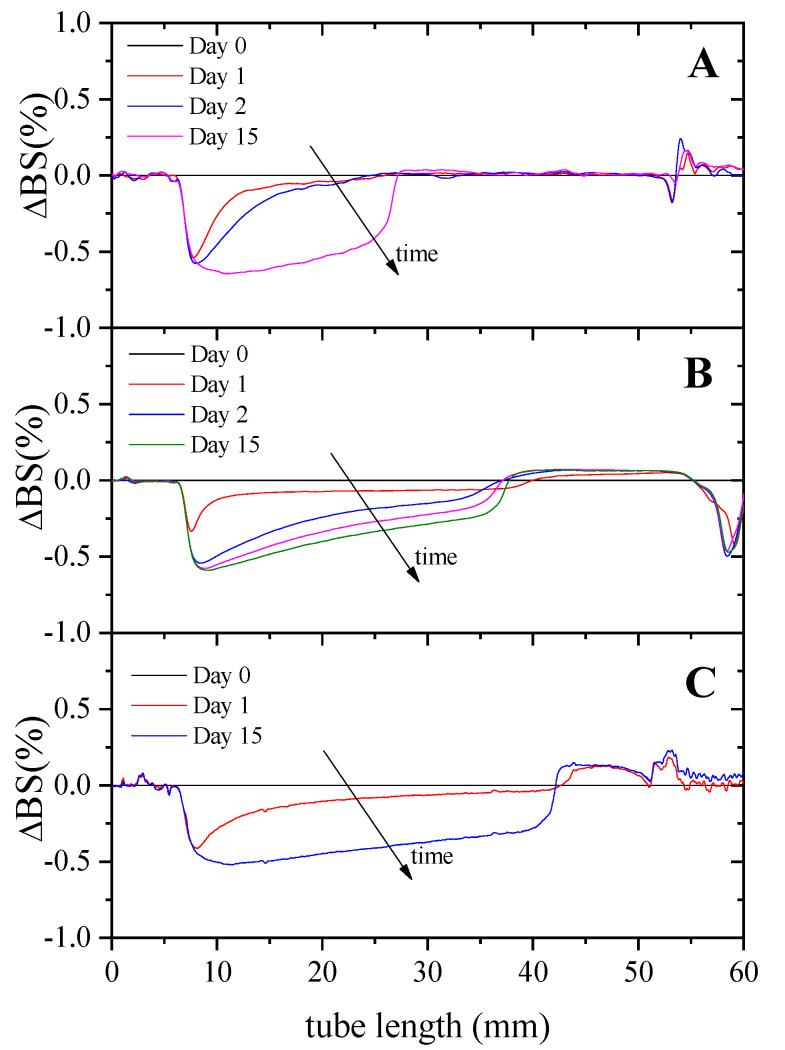
Changes in backscattering profiles (DBS%) as a function of sample height with storage time of W_1_/O/W_2_ multiple emulsions obtained after three different O/W ratios for the secondary emulsion: 40/60 (**A**), 25/75 (**B**) and 10/90 (**C**). The arrows indicate the storage time.

In order to analyze the stability of emulsions against creaming, Figure 4 shows the differential profiles of the backscattering variations (ΔBS%) when samples destabilize as a function of time.

At the beginning of the experiment, the backscattering of light was rather constant along with the entire height, as there was an even distribution of droplets throughout the system. Over time, droplets move upwards due to gravitational forces, which causes a decrease in the backscattering at the bottom of the emulsions (clarification) since the droplet concentration decreases. Conversely, an increase in the backscattering was detected at the top of the tube due to an increase in the droplet concentration (creaming). Clarification phenomenon is observed for all emulsions; however, creaming was only observed for the 10/90 and 25/75 systems.

#### 3.4.2. Rheological Characterization

**Figure 5:**
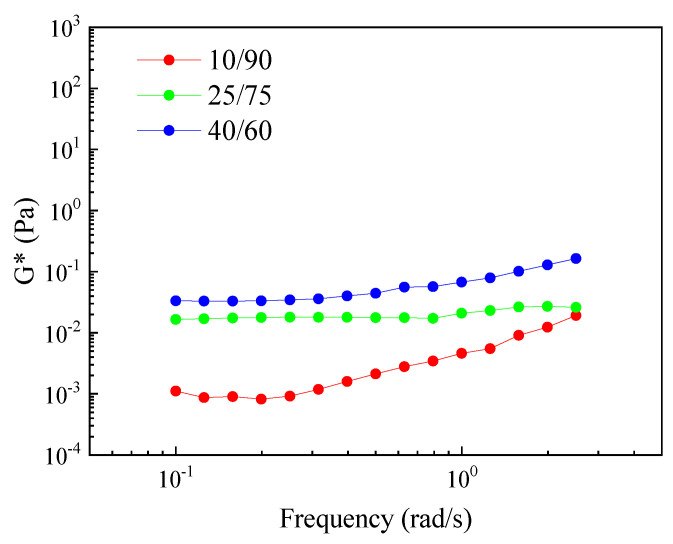
Complex moduli (G*) of multiple emulsions as a function of frequency obtained for three different (W_1_/O)/W_2_ ratios for the secondary emulsion: 10/90, 25/75 and 40/60.

Figure 5 shows complex moduli (G*) of multiple emulsions as a function of frequency obtained for three different (W_1_/O)/W_2_ ratios for the secondary emulsion: 10/90, 25/75 and 40/60.

This Figure shows an increase in the values of the complex modulus (G*) values when the proportion of the primary emulsion (W_1_/O) increases (from 10/90 to 40/60), which eventually results in an increase in the viscosity of the system. This same behavior has been previously found for conventional emulsions stabilized by proteins and was attributed to the formation of a network as a consequence of the closeness of the protein-stabilized oil droplets [31]. Moreover, the dependence of the complex moduli (G*) can also be analyzed, where an ideal gel-like response was related to a low dependence of G* with frequency [32]. Thus, the highest dependence of G* on frequency was observed for the system with the lowest W_1_/O content, indicating that this system not only had lower values of complex modulus but also that the system had a poorer rheological response.

Table 6 shows complex moduli at 0.2 Hz (G*_0.2_) as a function of time (1, 15 and 30 days) obtained for W_1_/O/W_2_ multiple emulsions at three different O/W ratios for the secondary emulsion (10/90, 25/75 and 40/60) to quantify the dependence of the G*_0.2_ on the concentration of the dispersed phase and the storage time.

This Table confirms a decrease in G* when the W_1_/O decreases, as well as a decrease in the value of G* over storage time for the three systems studied. The decrease in rheological functions with storage time has been previously observed for systems that suffered from destabilization, either by phase separation (creaming) or by coalescence [33]. In the systems studied in this work, the phenomenon of coalescence must be ruled out, as shown by the independence of the droplet size over time (Table 5). Thus, these results suggest that the structural stability of the systems could be increased by the addition of a hydrocolloid that structures the emulsions generated and prevent them from changing in their structure over storage time.

#### 3.4.3. Encapsulation Efficiency

Table 7 shows E_E_ as a function of time (1, 15 and 30 days) obtained for W_1_/O/W_2_ multiple emulsions at three different O/W ratios for the secondary emulsion (10/90, 25/75 and 40/60). This table shows suitable results in all cases where E_E_ was higher than 90% in all cases, regardless of the storage time. Only a slight release of the marker was seen in all systems.

These results would confirm the suitability of the processing conditions used for these emulsions since time emulsions did not suffer a significant decrease in this parameter after one-month storage. These results indicate that the formulation of these W_1_/O/W_2_ double emulsion systems in the three ratios studied allows the optimal encapsulation of the marker. Thus, the development of food matrices, in the form of multiple emulsions, would allow the encapsulation of bioactive compounds, which could result in the development of novelty food products.

### 3.5. Processing Conditions Optimization, Addition of Xanthan Gum

Up until now, the results indicate that the double emulsions generated had moderate stability together with an excellent E_E_. However, the structural stability of these systems could be increased by the addition of a hydrocolloid that would structure them and avoid destabilization phenomena such as emulsion creaming and further flocculation that could lead to unintended coalescence over storage time.

This section addresses the analysis of the microstructural characteristics of emulsions after the addition of xanthan gum (XG). It can be noticed that the E_E_ (%) of these systems was not determined since the addition of XG led to turbid emulsions after the centrifugation stage was carried out, which prevented reliable absorbance measurements. However, it is reasonable to assume that the E_E_ measures will not be affected by the addition of XG because it was dispersed by soft magnetic stirring after the double emulsion preparation.

#### 3.5.1. Droplet Size Distribution and Backscattering Measurements

Table 8 shows parameters from the DSD measurements (D_3,2_ and span) of W_1_/O/W_2_ multiple emulsions obtained at two different xanthan gum (XG) concentrations (0, 0.125 and 0.25 wt.%). These results agree with the results previously obtained for 40/60 (W_1_/O)/W_2_, confirming that the processing conditions followed did not influence the emulsions generated. Thus, the DSD parameters in Table 8 for the final 25/75 emulsions are practically the same as the parameters obtained for the 40/60 system (shown in Table 5) since these 25/75 double emulsions come from the dilution of 40/60 double emulsions. As for the influence of aging on these emulsions, this table shows that neither D_3,2_ nor Span parameters have undergone significant changes after one-month emulsion storage. The results indicated that the emulsions did not seem to be affected by coalescence phenomena over the storage time analyzed (which agrees with previous results) [26].

**Figure 6:**
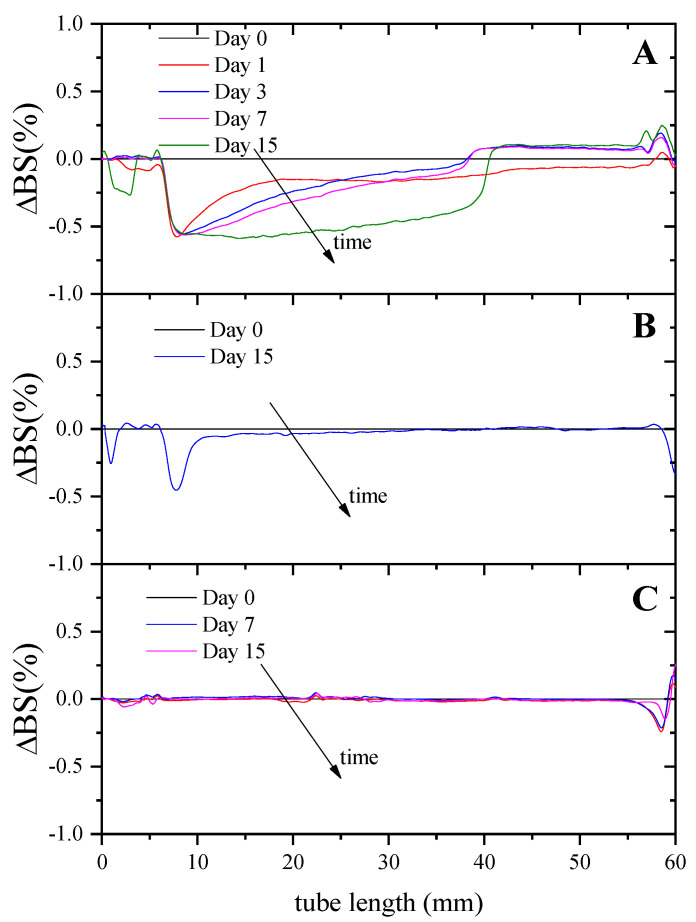
Backscattering profiles of W_1_/O/W_2_ multiple emulsions obtained at two different xanthan gum (XG) concentrations: 0 (**A**), 0.125 (**B**) and 0.25 (**C**) wt.%.

In order to analyze the stability of these emulsions against creaming, Figure 6 shows the changes in backscattering profiles (ΔBS%) at two different XG concentrations (0, 0.125 and 0.25 wt.%). These graphs show that an increase in XG concentration gives rise to an increase in stability against creaming; this phenomenon disappeared completely at the highest concentration studied (0.25% XG). These results indicate that the addition of the hydrocolloid causes an increase in stability as a consequence of the structuring of the system [34].

Previous results indicated the occurrence of microstructural changes in these emulsions. Consequently, rheological tests were carried out to investigate if the addition of XG reduces it. Bulk rheology helps the understanding of the physical origin of emulsion creaming and flocculation [35].

#### 3.5.2. Rheological Characterization

**Figure 7:**
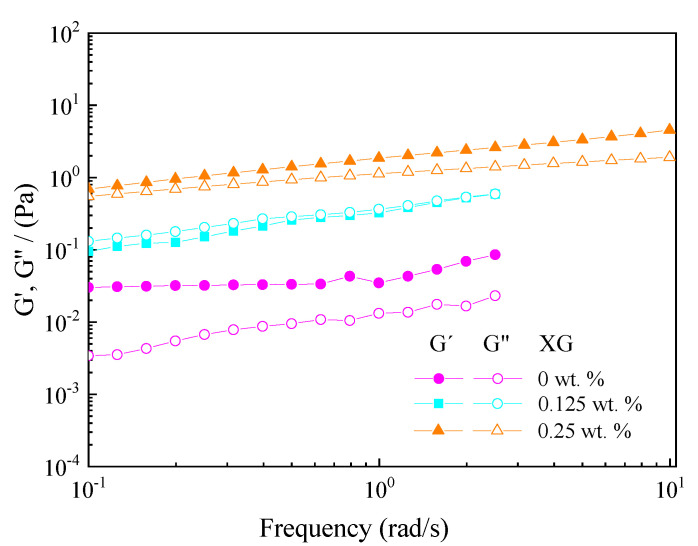
Viscoelastic moduli (G′ and G″) as a function of frequency of W_1_/O/W_2_ multiple emulsions obtained at three different xanthan gum (XG) concentrations (0, 0.125 and 0.25 wt.%) obtained the same day emulsion preparation.

Figure 7 shows viscoelastic moduli (G′ and G″) of W_1_/O/W_2_ multiple emulsions as a function of frequency obtained at three different XG concentrations (0, 0.125 and 0.25 wt.%) in the same day emulsion preparation. The rheological response obtained corresponds to a weak gel-like structure, where the elastic component is predominant (G′ > G″), even for the lowest XG concentration. This response has been previously observed for diluted emulsions (which is the case) due to the lack of emulsion flocculation [28].

The influence of XG on structuring the W_1_/O/W_2_ multiple emulsions generated is corroborated since an increase in XG concentration involved an increase of the viscoelastic moduli (G′ and G″). Thus, the emulsions with the lowest XG concentration exhibited the weakest microstructure (lower viscoelastic moduli), and the emulsion with the highest XG concentration exhibited the highest G′ and G″ values. The gel-like behavior of emulsions has been attributed either to the packing effect of droplets surrounded by proteins and/or the formation of a biopolymer network [36].

## 4. Conclusions

The results show that multiple W_1_/O/W_2_ emulsions can be obtained after selecting suitable processing conditions, which in turn, makes them easily scalable for industrial processes. This has great importance since these emulsions could be used for encapsulation of both hydrophilic and lipophilic components. The analysis of the processing conditions showed that several passes of the emulsion through the valve homogenizer led to narrower droplet size distributions. However, excessive processing resulted in droplet coalescence due to an excess of energy input, which involved larger droplet sizes. Thus, in terms of droplet size distribution, the input of higher energy to the system (20 MPa) during emulsification processing led to emulsions with smaller droplet sizes (D_3,2_). However, more monodispersed emulsions were achieved when the lowest pressure (5 MPa) was used. As for the number of passes, the optimal (emulsions more monodispersed and smaller droplet sizes) was found around 2–3 passes regardless of the valve homogenizer pressure. However, emulsions processed at 20 MPa involved lower EE values than emulsions processed at 5 MPa (87.3 ± 2.3 vs. 96.1 ± 1.8, respectively).

Although microstructural properties of emulsions can be modulated by the ratio between the primary and secondary emulsions ((W_1_/O)/W_2_ ratio), all the emulsions generated without XG presented similar stability and EE (~95%). This result suggests that different products exhibiting different textures (i.e., mayonnaise, salad dressings, milkshake, etc.) could be developed based on these emulsions. However, emulsions without XG resulted in a higher destabilization over storage time.

These results indicate that the formulation of these W1/O/W2 double emulsion systems allows the optimal encapsulation of the marker. Thus, the development of food matrices, in the form of multiple emulsions, would allow the encapsulation of bioactive compounds, which could result in the development of novelty food products.

## Figures and Tables

**Table 1:** Marker concentration measurable (MC_m_) and recovery yield (Ry) for the marker (tartrazine) in the aqueous phase recovered standard where 100% of the marker is in the outer aqueous phase (W_2_). Different letters within a row indicate significant differences (*p* < 0.05).

Day	MC_m_ (%)	Ry (%)
0	100.0 ± 0.5 ^a^	95.5 ± 1.5 ^a^
7	98.8 ± 0.2 ^b^	98.9 ± 0.2 ^a^

**Table 2:** Parameters (D_3,2_, Span and FI) from droplet size distribution as a function of the number of emulsion passes through the high-pressure homogenizer and the two pressures studied (5 MPa and 20 MPa). Different superscript letters within a column indicate significant differences (*p* < 0.05).

Passes	5 MPa			20 MPa		
	D_3,2_ (μm)	Span	FI (%)	D_3,2_ (μm)	Span	FI (%)
1	5.2 ± 0.3 ^a^	2.2 ± 0.1 ^a^	5.2 ± 3.2 ^a^	4.1 ± 0.3 ^a^	1.9 ± 0.4 ^a^	20.0 ± 8.0 ^a^
2	4.7 ± 0.2 ^b^	1.8 ± 0.1 ^b^	9.5 ± 2.1 ^b^	2.3 ± 0.2 ^b^	2.2 ± 0.3 ^a^	13.2 ± 6.8 ^b^
3	4.0 ± 0.1 ^c^	1.6 ± 0.2 ^c^	3.6 ± 2.2 ^a^	1.9 ± 0.2 ^b^	2.6 ± 0.2 ^b^	6.4 ± 4.2 ^c^
4	4.3 ± 0.2 ^c^	1.4 ± 0.1 ^c^	14.0 ± 4.1 ^c^	1.9 ± 0.1 ^b^	2.2 ± 0.3 ^a^	6.0 ± 4.0 ^c^
5	4.5 ± 0.2 ^c,d^	1.2 ± 0.1 ^d^	9.0 ± 2.9 ^b^	2.0 ± 0.3 ^b^	4.0 ± 0.2 ^c^	28.0 ± 8.2 ^a^

**Table 3:** Encapsulation efficiency (E_E_) for the emulsions as a function of the number of emulsion passes through the high-pressure homogenizer (steps) and the two pressures studied (5 MPa and 20 MPa). Different superscript letters within a column indicate significant differences (*p* < 0.05).

Passes	E_E_ (%) 5 MPa	E_E_ (%) 20 MPa
1	97.0 ± 2.1 ^a^	86.1 ± 3.1 ^a^
2	97.6 ± 1.2 ^a^	54.1 ± 6.1 ^b^
3	96.1 ± 1.8 ^a^	87.3 ± 2.3 ^a^
4	95.4 ± 1.5 ^a^	74.3 ± 5.6 ^c^
5	97.0 ± 1.1 ^a^	87.3 ± 2.9 ^a^

**Table 4:** Parameters from droplet size distribution (D_3,2_, Span and FI) as well as encapsulation efficiency (E_E_) at three different PGPR concentrations (1, 3 and 5 wt.%). Different superscript letters within a column indicate significant differences (*p* < 0.05).

% PGPR	D_3,2_ (μm)	Span	FI (%)	E_E_ (%)
1	2.9 ± 0.1 ^a^	1.9 ± 0.2 ^a^	15.2 ± 8.2 ^a^	69.1 ± 4.1 ^a^
3	4.3 ± 0.1 ^b^	1.5 ± 0.2 ^b^	16.8 ± 9.3 ^a^	84.1 ± 3.8 ^b^
5	4.0 ± 0.1 ^b^	1.6 ± 0.2 ^b^	3.6 ± 2.2 ^b^	96.1 ± 1.8 ^c^

**Table 5:** Parameters from droplet size distribution (D_3,2_, span and FI) of W_1_/O/W_2_ multiple emulsions obtained after evaluating three different O/W ratios (10/90, 25/75 and 40/60) for the secondary emulsion over 30 days storage time. Different superscript letters within a column indicate significant differences (*p* < 0.05).

Day	10/90			25/75			40/60		
	D_3,2_ (μm)	Span	FI (%)	D_3,2_ (μm)	Span	FI (%)	D_3,2_ (μm)	Span	FI (%)
0	4.1 ± 0.2 ^a^	1.8 ± 0.2 ^a^	10.2 ± 3.8 ^a^	4.8 ± 0.2 ^a^	1.8 ± 0.3 ^a^	7.8 ± 3.1 ^a^	5.8 ± 0.2 ^a^	1.9 ± 0.2 ^a^	13.7 ± 5.0 ^a^
1	4.2 ± 0.1 ^a^	1.7 ± 0.2 ^a^	12.1 ± 3.1 ^a^	4.6 ± 0.1 ^a^	1.6 ± 0.2 ^a^	12.2 ± 4.0 ^b^	5.6 ± 0.1 ^a^	2.1 ± 0.2 ^a^	10.3 ± 4.1 ^a^
15	4.4 ± 0.1 ^a^	1.8 ± 0.1 ^a^	6.9 ± 4.1 ^b^	5.0 ± 0.2 ^a^	1.7± 0.3 ^a^	11.1 ± 4.2 ^b^	5.6 ± 0.2 ^a^	2.1 ± 0.1 ^a^	6.1 ± 3.2 ^b^
30	3.8 ± 0.3 ^a^	1.8 ± 0.2 ^a^	7.4 ± 2.7 ^b^	4.9 ± 0.2 ^a^	1.8 ± 0.1 ^a^	12.6 ± 5.2 ^b^	5.8 ± 0.1 ^a^	1.7 ± 0.2 ^b^	9.7 ± 3.7 ^b^

**Table 6:** Complex modulus at 0.2 Hz (G*_0.2_) as a function of time (1, 15 and 30 days) obtained for W_1_/O/W_2_ multiple emulsions at three different O/W ratios for the secondary emulsion (10/90, 25/75 and 40/60). Different superscript letters within a column indicate significant differences (*p* < 0.05).

Day	E_E_ (%)		
	10/90	25/75	60/40
1	93.3 ± 2.0 ^a^	99.8 ± 0.2 ^a^	97.2 ± 1.1 ^a^
15	95.2 ± 2.5 ^a^	95.0 ± 1.2 ^b^	94.7 ± 1.5 ^b^
30	93.0 ± 3.1 ^a^	91.0 ± 2.0 ^c^	95.0 ± 1.2 ^b^

**Table 7:** Encapsulation efficiency (E_E_) as a function of time (1, 15 and 30 days) obtained for W_1_/O/W_2_ multiple emulsions at three different O/W ratios for the secondary emulsion (10/90, 25/75 and 40/60). Different superscript letters within a column indicate significant differences (*p* < 0.05).

Day	G*_0.2_ (Pa)		
	10/90	25/75	40/60
1	0.023 ± 0.002 ^a^	0.055 ± 0.002 ^a^	0.079 ± 0.001 ^a^
15	0.015 ± 0.003 ^b^	0.038 ± 0.004 ^b^	0.026 ± 0.003 ^b^
30	0.014 ± 0.002 ^c^	0.015 ^c^ ± 0.002 ^c^	0.025 ± 0.002 ^b^

**Table 8:** Parameters from Droplet size distribution (D_3,2_ and span) of W_1_/O/W_2_ multiple emulsions obtained at two different xanthan gum (XG) concentrations: 0, 0.125 and 0.25 wt.%.

Day	0 wt.% XG		0.125 wt.% XG		0.25 wt.% XG	
	D_3,2_ (μm)	Span	D_3,2_ (μm)	Span	D_3,2_ (μm)	Span
0	4.3 ± 0.2 ^a^	1.6 ± 0.2 ^a^	4.3 ± 0.2 ^a^	1.5 ± 0.3 ^a^	4.1 ± 0.2 ^a^	1.7 ± 0.3 ^a^
1	4.6 ± 0.1 ^a^	1.7 ± 0.3 ^a^	4.0 ± 0.3 ^a^	1.6 ± 0.3 ^a^	4.1 ± 0.3 ^a^	1.8 ± 0.2 ^a^
15	4.0 ± 0.3 ^a^	1.5 ± 0.3 ^a^	3.9 ± 0.3 ^a^	1.6 ± 0.2 ^a^	3.8 ± 0.4 ^a^	1.7 ± 0.3 ^a^
30	3.9 ± 0.3 ^a^	1.8 ± 0.3 ^a^	3.8 ± 0.4 ^a^	2.0 ± 0.2 ^b^	3.8 ± 0.3 ^a^	2.0 ± 0.3 ^a^

## Data Availability

The datasets generated for this study are available on request to the corresponding author.

## Supplementary Materials

The following supporting information can be downloaded at: https://www.mdpi.com/article/10.3390/foods11091367/s1, Table S1. Correlation of systems analyzed in this research, evaluating two parameters: the concentration of the oil phase ratio in the secondary emulsion as well as the effect of the thickening agent. Figure S1. Images of double emulsion droplets (emulsion containing 25/75 of (W_1_/O)/W_2_) obtained from optical microscopy.
