# Supplementary material for: Optimization of Multiple W1/O/W2 Emulsions Processing for Suitable Stability and Encapsulation Efficiency
Source: Foods. 2022 May 9;11(9):1367. doi: 10.3390/foods11091367 (PMC9103989; doi:10.3390/foods11091367)
Supplement: Supplementary file 1 [file foods-11-01367-s001.zip › foods-1686534-supplementary.pdf]

Supplementary Material

Table S1. Correlation of systems analysed in this research, evaluating two parameters: the concentration of the oil phase ratio in the secondary emulsion as well as the effect of the thickening agent.

Passes	Evaluation of Oil Phase	Evaluation of Thickenning Agent
	10/90	0
**$(W_1/O)/W_2$ ratio**	25/75	0
	60/40	0
	25/75	0
**XG concentration (wt.%)**	25/75	1.25
	25/75	0.25

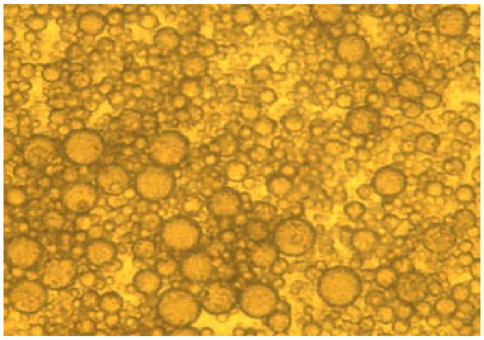

Figure S1. Images of double emulsion droplets (emulsion containing 25/75 of (W1/O)/W2) obtained from an optical microscopy.
